# SkeletalVis: an exploration and meta-analysis data portal of cross-species skeletal transcriptomics data

**DOI:** 10.1093/bioinformatics/bty947

**Published:** 2018-11-27

**Authors:** Jamie Soul, Tim E Hardingham, Ray P Boot-Handford, Jean-Marc Schwartz

**Affiliations:** 1 Division of Evolution & Genomic Sciences, University of Manchester, Manchester, MUK; 2 Wellcome Centre for Cell-Matrix Research, Division of Cell-Matrix Biology and Regenerative Medicine, Faculty of Biology Medicine and Health, University of Manchester, Manchester, MUK

## Abstract

**Motivation:**

Skeletal diseases are prevalent in society, but improved molecular understanding is required to formulate new therapeutic strategies. Large and increasing quantities of available skeletal transcriptomics experiments give the potential for mechanistic insight of both fundamental skeletal biology and skeletal disease. However, no current repository provides access to processed, readily interpretable analysis of this data. To address this, we have developed SkeletalVis, an exploration portal for skeletal gene expression experiments.

**Results:**

The SkeletalVis data portal provides an exploration and comparison platform for analysed skeletal transcriptomics data. It currently hosts 287 analysed experiments with 739 perturbation responses with comprehensive downstream analysis. We demonstrate its utility in identifying both known and novel relationships between skeletal expression signatures. SkeletalVis provides users with a platform to explore the wealth of available expression data, develop consensus signatures and the ability to compare gene signatures from new experiments to the analysed data to facilitate meta-analysis.

**Availability and implementation:**

The SkeletalVis data portal is freely accessible at http://phenome.manchester.ac.uk.

**Supplementary information:**

Supplementary data are available at *Bioinformatics* online.

## 1 Introduction

Skeletal disease affects millions of the adult population, causing a huge burden on healthcare systems (Cross *et al.*, 2014). This includes common polygenic forms of joint disease such as osteoarthritis (OA) and rheumatoid arthritis (RA) and the rare monogenic skeletal conditions. Despite years of research there are no disease modifying drugs for osteoarthritis and other skeletal diseases (Karsdal *et al.*, 2016). There is a critical need to understand the underlying molecular mechanisms to find potential therapeutic targets. Transcriptomics analysis of diseased cells or tissues gives us insight into altered expression of genes which are potentially causing an individual disease. There is a large and growing amount of publicly available expression data from microarray and more recently RNA-Seq for skeletal disease (Steinberg and Zeggini, 2016). These datasets are analysed to produce lists of differentially expressed genes and derive broader functional information such as enriched pathways. For instance, we have previously used transcriptomics data to understand the altered processes in osteoarthritis cartilage damage and other researchers have characterized mouse models of rare skeletal diseases using transcriptomics (Cameron *et al.*, 2011; Dunn *et al.*, 2016; Soul *et al.*, 2018). Despite the use of global transcriptomics, papers describing these experiments only focus on a fraction of the information within these datasets. Extended use of this existing data would allow exploration and mining for new overlooked features. With the increased coverage of annotations for pathways/transcription factors and improved methods of analysis, re-analysis of these datasets may identify new features (Wadi *et al.*, 2016). Furthermore, consistent analysis and integration of the data would allow identification of similarities between new and existing datasets, allowing for sharing of knowledge between diseases and experimental models, identification of shared pathogenic mechanisms and giving the potential for re-purposing of therapeutics and identification of new experimental models.

The differentially expressed genes and downstream analysis (e.g. enriched pathways) generated from an experiment generally only exist as Supplementary Tables in the original publication creating a barrier to the reuse and integration of these datasets. Furthermore, the inconsistent methods used in analysis of the published transcriptomics data render robust direct comparison of datasets challenging. An increasing number of researchers are depositing their raw transcriptomic data in public transcriptomics repositories such as ArrayExpress and GEO that provide the transcriptomics data and meta-data needed to re-analyse the data (Edgar, 2002; Kolesnikov *et al.*, 2015).The EBI Expression Atlas has begun to analyse existing transcriptomics data and offers exploration of selected public datasets with differential expression analysis and basic pathway analysis (Papatheodorou *et al.*, 2018). However, as this database is unfocused on a particular area of biology it has poor coverage of the available skeletal disease data and offers no way to compare expression responses between experiments or generate consensus signatures for further analysis. The CREEDS portal allows comparison of query gene expression signatures against a large database of GEO-derived signatures, but does not allow assessment of the quality and exploration of the underlying datasets (Wang *et al.*, 2016). Furthermore, this search engine relies on automatic identification of the perturbation and the control samples, which although very scalable, is less accurate than human curation. As with Expression Atlas there is poor coverage of skeletal disease-related datasets.

With the growing repository of skeletal disease transcriptomic data available there is now the opportunity to systematically analyse and integrate this data. Specialized repositories exist for diseases such as cancer, but none currently exist for skeletal disease to make use of this data (Bowman *et al.*, 2017). We have therefore developed a web-application to allow exploration and comparisons of publicly available skeletal disease transcriptomics data in order to analyse the pathology and predict the active mechanisms driving skeletal disease. The SkeletalVis data-portal avoids the requirement for bench scientists to download raw data and re-analyse every dataset needed in a comparison. We highlight its utility in exploring this data, identifying the similarities between skeletal disease models and skeletal genetic perturbations and elucidation of potential therapeutic targets for groups of similar expression responses. The SkeletalVis data portal is freely available at phenome.manchester.ac.uk.

## 2 Materials and methods

### 2.1 Identification and annotation of skeletal transcriptomic datasets

The ArrayExpress and GEO databases and the linked European nucleotide archive (ENA) and sequence read archive (SRA) data repositories were searched for keywords relating to skeletal cell types and skeletal disease (Supplementary Table S1) (Leinonen *et al.*, 2011a,b). These results were filtered to keep only those experiments using whole genome mRNA transcriptomics with raw data available for the commonly studied species of Cow, Human, Mouse, Pig and Rat. Experiments were annotated by the experimental platform, the tissue under study, the type of experimental perturbation and with a concise description of the experiment. Comparisons (contrasts) to perform within each experiment were identified manually through the provided meta-data and corresponding publication (if available) for each experiment.

### 2.2 Transcriptomics analysis pipeline

A Galaxy pipeline was used to analyse the identified experiments in a high-throughput manner. Where available existing tools were used from Galaxy tool-shed, otherwise RGalaxy (v1.22.0) was used to create bespoke tools (Afgan *et al.*, 2016). These modules were linked together to create a flexible pipeline for the analysis of microarray or RNA-seq data. For RNA-seq the raw data and meta-data were downloaded from ENA/SRA. Pseudo-alignment and qualification of reads was performed with Kallisto (v0.43.0) using the Ensembl transcriptome reference (release 79) for the appropriate species (Bray *et al.*, 2016). MultiQC (v1.4) was used to generate summary reports of the FastQC (v0.11.5) read statistics and Kallisto mapping logs for quality control (Ewels *et al.*, 2016). Tximport (v1.6.0) was used to summarize the mapped transcript level counts to gene level (Soneson *et al.*, 2016). For microarray experiments, the unnormalized data and meta-data were downloaded from GEO or ArrayExpress. Normalization was performed using the robust mean average for Affymetrix arrays and quantile normalization for Illumina and Agilent arrays. Probesets were collapsed to the median value to provide a representative level of expression. Poor quality samples that are either mentioned in the corresponding publication or based on quality control data were removed to ensure robust gene expression signatures.

Batch effect has previous been established as a confounding factor in differential expression analysis. For both RNA-seq and microarray data, unless experimental batches were explicitly stated in the experimental meta-data they were inferred using sva (v3.26.0) with automatic identification of the number of surrogate variables (Leek *et al.*, 2012). For visualization of the experimental samples by principal component analysis (PCA) the surrogate variables were regressed from the expression matrix before PCA. Limma (v3.34.9) and DESeq2 (v1.18.1) were used to calculate fold-changes and *P*-values between the comparisons in each experiment for microarray and RNA-Seq experiments, respectively (Love *et al.*, 2014; Ritchie *et al.*, 2015). Where insufficient replicates (<3) were available in an experiment only the fold-changes were calculated. The surrogate variables were incorporated as co-variants in the limma or DESeq2 statistical models to correct for the identified batch effects. Independent filtering based on mean gene-wise microarray intensity or RNA-seq read count was used to minimize false-positive differentially expressed genes, as implemented in the genefilter package (the default procedure in DESeq2), with automatic selection of the expression filtering threshold based on the number of differentially expressed genes (Bourgon *et al.*, 2010). Benjamini-Hochberg correction was performed on the resulting *P*-values to account for multiple testing. As an alternative method for identifying differentially expressed genes, the characteristic direction method as implemented in GeoDE (v1.0) was used with the batch effect corrected count/intensity matrix using a threshold of the top 500 influential genes as previously used (Clark *et al.*, 2014). As a quality control check, where published, the transcriptomics results were checked to ensure they were broadly similar to the re-analysed data with differentially expressed genes mentioned in the corresponding publication dysregulated in our analysis.

Differentially expressed genes from RNA-seq and microarray experiments were further analysed in a common downstream pipeline. For enrichment-based methods, differentially expressed genes were defined with combinations of absolute fold-change (none, 1.5 or 2) and an adjusted *P*-value (none, 0.05) thresholds. Pathway (PathwayCommons) and gene ontology biological process (GOBP) enrichment was performed using goseq (v1.30.0) (Ashburner *et al.*, 2000; Cerami *et al.*, 2011; Young *et al.*, 2010. Significant pathways and GOBP terms were defined with an adjusted *P*-value ≤ 0.05 threshold. Redundancy of the pathways was reduced with the set cover algorithm (Stoney *et al.*, 2018). Redundancy reduction of the GOBP terms was performed using the Revigo algorithm with the Resnick semantic similarity threshold set to 0.4 (Supek *et al.*, 2011). Significantly enriched transcription factors based on motif occurrence in the differentially expressed genes were identified by RcisTarget (v0.99.0) for mouse and human experiments (Aibar *et al.*, 2017).

For network analysis the human STRINGDB (v10.5) and the BioGrid (v3.4.162) protein-protein interaction networks were used with Ensembl ortholog mapping for other species. The STRINGDB network was filtered using an edge confidence threshold of > 400 to remove low quality interactions and text-mining derived edges were removed. The largest connected components were retained for both networks (von Mering *et al.*, 2003). Active sub-networks (de novo pathways) were identified using the ranked list of differential expression data and the GIGA algorithm with a maximum sub-network size of 10 (Breitling *et al.*, 2004). GO enrichment of the genes in the sub-network was used to identify the function of the sub-network.

To identify potential drugs that could reverse or mimic the observed differential expression the LINCS L1000 perturbation database, accessed with the L1000CDS2 API, was used to find overlap between the gene expression signatures (Duan *et al.*, 2016). To annotate molecular targets of the enriched drugs the PubChem BioAssay Database was queried to find proteins which each drug has activity against (Wang *et al.*, 2012).

Code for the pipeline and post-processing of the data can be found at www.github.com/soulj/SkeletalVis-Pipeline

### 2.3 Expression similarity

To allow comparison of the gene expression across species, all genes symbols were mapped to human gene symbols using Ensembl orthologs. Genes not measured in an experiment were regarded as NA. Four measures of gene expression similarity were calculated to allow comparison which considers the direction of the fold-change.

The signed Jaccard index for two signatures $\;S_{i\;}$ and $\;S_{j\;}$ is defined as:

$$SJ\left(S_{i},S_{j}\right)=\frac{J\left(S_{i}^{\text{up}},S_{j}^{\text{up}}\right)+J\left(S_{i}^{\text{down}},S_{j}^{\text{down}}\right)-J\left(S_{i}^{\text{up}},S_{j}^{\text{down}}\right)-J\left(S_{i}^{\text{down}},S_{j}^{\text{up}}\right)}{2}$$

where $\;S^{\text{up}}$ and $S^{\text{down}}$ refer to the up- and down-regulated genes respectively.

This measure was calculated with the gene expression signatures defined with (i) a 1.5-fold change threshold, (ii) a 1.5-fold change threshold and an adjusted *P*-value ≤ 0.05 threshold and (iii) the characteristic direction genes.

The cosine similarity measure (the cosine of the angle between two expression vectors) was also calculated using the fold-changes of each perturbation response as an alternative to the set overlap-based measures.

### 2.4 t-Distributed stochastic neighbour embedding visualization of signature similarity

t-Distributed stochastic neighbour embedding (t-SNE) implemented in Rtsne (v0.13) was run 1000 times with a perplexity of 10 and the clustering solution with the lowest KL divergence was selected (Maaten and Hinton, 2008). dbscan (v1.1.1) clustering with the size of the epsilon neighbourhood set to 2.5 was used to colour groups of density in the plot (Ester *et al.*, 1996). The groups were labelled based on representative descriptions of the perturbations within the groups.

### 2.5 Identification of perturbation group consensus signatures and enriched drugs

Consensus signatures were generated for each of the t-SNE perturbation groups by applying the RankProd (v3.4) rank product method to perturbation gene log ratios in each group (Del Carratore *et al.*, 2017). Genes with a percentage of false-positive predictions ≤ 0.05 were used to identify enriched mimic and reverse drugs signatures using the LINCS L1000 drug signatures as described earlier.

### 2.6 Web interface

SkeletalVis is an interactive web-based tool which can run on any common browser such as Chrome, Firefox and Safari. The web application is implemented using the R Shiny framework designed to display the output of the Galaxy pipeline as well as the processed expression similarity data. The application makes use of datatables (v0.2) for responsive tables to allowing fluid data exploration and enrichR (v1.0) to identify enriched pathways from user generated consensus signatures. Interactive visualization of graphs and networks was implemented with plotly (v4.7.1) and visNetworks (2.0.1). Single, global loading of the data ensures a responsive application. Code for the web interface can be found at www.github.com/soulj/SkeletalVis-Shiny

## 3 Results

### 3.1 Re-analysis of skeletal transcriptomics data

**Fig. 1.**
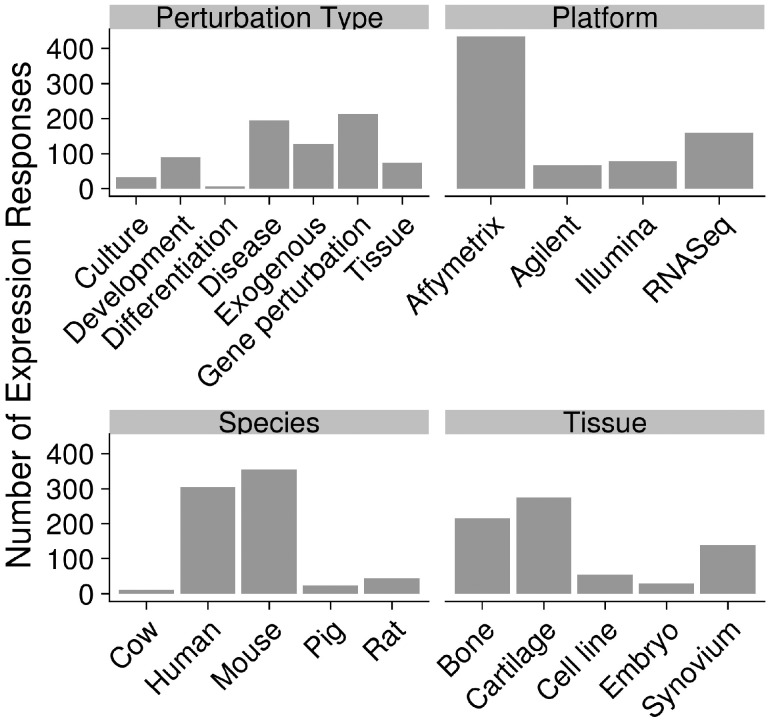
Summary of analysed expression perturbations

Our overall approach was to use a high-throughput, transcriptomics pipeline to analyse existing skeletal disease transcriptomics data. Searching for relevant datasets in ArrayExpress and GEO identified 287 experiments (Supplementary Table S2). Analysis of the raw data through the transcriptomics pipeline generated 739 expression response profiles with quality control and PCA plots, differential expression and comprehensive downstream analysis comprising of pathway, active sub-network, GO Term, drug, transcription factor enrichment (Supplementary Fig. S1). Annotation of these datasets revealed a variety of platforms, species and experimental design types (Fig. 1). The majority of datasets was from human and mouse reflecting the focus on use of both human tissue and mouse models for the study of skeletal disease and showing need for cross-species analysis. Affymetrix was the most common array type and there is a growing number of RNA-Seq (Illumina)-based datasets.

### 3.2 Recovery of prior knowledge and assessment of bias

Expression similarity is a useful tool to find related biological responses to perturbations thereby identifying diseases that potentially share similar mechanisms. A recurrent barrier to this type of analysis is the heterogeneous experimental platforms used to measure the gene expression (Leek *et al.*, 2012). To investigate wherever there is strong bias in expression similarity of the analysed comparisons due to the experimental platform, we compared the similarity rank of experiments within the same platform compared with different platforms (Supplementary Fig. S2A). No strong global bias was observed in the similarity of perturbations within platforms. Likewise, no strong global similarity between perturbations within the tissue under study was observed, but a stronger bias was seen between species, possibly due to the nature of the experiments performed in the different species (Supplementary Fig. S2B and C).

To validate the expression similarity analysis, sets of experiments examining well-defined, related experimental perturbations that would be expected to have a shared expression response were selected i.e. same gene perturbation, exogenous treatment or disease (Supplementary Table S3). For each set of experiments the rank based on the four genes expression similarity measures (see methods) of the annotated experiments was calculated (Supplementary Fig. S3). Despite differences in the experimental set-up of the related experiments, the annotated datasets were among the most similar in the database, suggesting we can recover prior biological knowledge. The characteristic direction measure showed the best performance with these datasets consistent with a previous assessment against limma-based differential expression analysis (Wang *et al.*, 2016). These findings suggest that the strength of the biological signal from the perturbations is sufficient to identify related experiments with shared biological mechanisms.

### 3.3 Identifying signature associations in skeletal biology

Associations between single gene perturbations and other transcriptomic responses can imply upstream regulation or shared signalling cascades. Transcriptomic signatures from gene perturbation experiments were examined to find the top pairwise similar expression responses to highlight examples of association identification enabled by this re-analysis of the transcriptomic datasets (Supplementary Table S4). Several examples which demonstrate cross-species *in vitro* and *in vivo* response similarity are shown (Table 1). The perturbation signature from mouse Eed knockout in rib cartilage shows similarity to mouse Ezh2 knockout growth plate zones, both components are part of the Polycomb repressive Complex 2 which methylates target genes (Mirzamohammadi *et al.*, 2016; Lui *et al.*, 2016). The Eed knockout signature also shows similarity to the *in vitro* human chondrocyte de-differentiation expression responses. The androgen receptor knockout mice signature is similar to the oestrogen receptor knockout (Kondoh *et al.*, 2014; Russell *et al.*, 2012). Interestingly, two skeletal disease mouse models also show similarity to the androgen receptor knockout; the Phex-deficient Hyp mouse model of X-linked hypophosphatemia and the AHSG knockout model of model of slipped capital femoral epiphysis (SCFE) (Brylka *et al.*, 2017). Likewise, inhibition of the DOTL1 methyltransferase shows similarity to several inflammatory datasets (Monteagudo *et al.*, 2017). These results suggest that by examining the similarities between cross-species skeletal transcriptomic responses from gene perturbations we can both recover known relationships and identify novel associations for future experimental validation.

**Table 1.** The top pairwise similar characteristic direction expression responses for selected gene perturbations. Unless stated perturbations are relative to wild type/control conditions

Perturbation	Accession	Species	Signed Jaccard
Eed knockout	GSE66862	Mouse	
Ezh knockout	GSE84198	Mouse	0.0574
Superficial versus deep zone cartilage	E-GEOD-54216	Rat	0.0563
Dedifferentiating chondrocytes	GSE42235	Human	0.0447
mOL-AR knockout	E-MTAB-1123	Mouse	
AhsgHET versus Ahsg WT	GSE105139	Mouse	0.0579
Hyp females versus Wildtype females	GSE5657	Mouse	0.0574
Estrogen Receptor alpha knock-out	GSE41997	Mouse	0.0495
DOT1L inhibition	GSE77916	Human	
Galectin 1 treatment	E-GEOD-68760	Human	0.0400
Galectin3 treatment	GSE85254	Human	0.0315
IL-1 and glucosamine treatment	E-GEOD-6119	Rat	0.0311

**Fig. 2.**
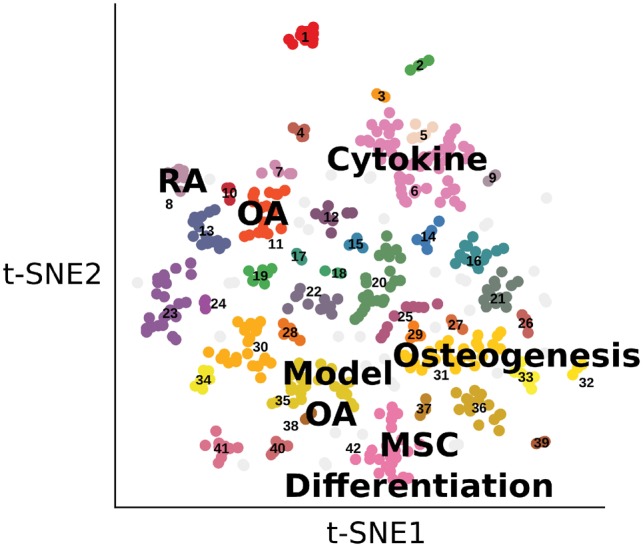
t-SNE visualization of skeletal expression signatures using the characteristic direction signature distance matrix. Groups of perturbations are labelled and coloured by regions of density identified using dbscan

To further explore the gene expression signatures we applied the t-SNE algorithm to visualize the characteristic direction derived signatures distance matrix, enabling a global overview of the skeletal disease transcriptional landscape and exploration of groups of related experimental perturbations. The resulting plot shows the heterogeneous perturbations broadly separate into groups of related perturbations with similar expression responses (Fig. 2). Several of the groups demonstrate the ability of this analysis to highlight-related experiments (Supplementary Table S5). For instance, several rheumatoid arthritis datasets are clustered together (Group 8). Similarly many short-term cytokine stimulated tissue perturbations form a group (Group 6).This analysis also highlights the ability to identify cross-species groups of profiles. For example, the model OA group (Group 35) includes a diverse collection of cross-species osteoarthritis animal model perturbations such as the mouse surgical destabilization of the medial meniscus post-traumatic model of OA and the rat metabolic model of OA with monoiodoacetate treatment (Burleigh *et al.*, 2012; Korostynski *et al.*, 2018; Loeser *et al.*, 2013).These results suggest similarity in the expression response and shared mechanisms of action in these different models of induced osteoarthritis leading to the degradation of cartilage.

To identify compounds potentially capable of mimicking or reversing the observed differential expression in the identified perturbation groups, we generated rank-product consensus signatures and performed drug enrichment analysis (Supplementary Tables S6–S8). A group of *in vitro* histone deacetylase (HDAC) inhibitor perturbations corresponded with several HDAC inhibitor drug signatures (Table 2). An activator of PKC, part of the cytokine signalling cascade, was predicted to mimic the cytokine stimulation responses. Several drugs were found to have an opposite transcriptomic response in skeletal disease-related groups. For instance, in the model osteoarthritis group MEK1/2 and PI3K inhibitors were identified among the top reverse drug signatures. These results suggest we can generate robust consensus signatures from groups of transcriptomic signatures to both recover known signalling pathways and predict potential therapeutic targets.

**Table 2.** Top mimic or reverse drugs for selected perturbation groups. Enriched drugs with nominal targets and overlap scores were found using the LINCSL1000 CDS database for the t-SNE perturbation group consensus signatures. The enriched drugs act as inhibitors of the indicated targets unless otherwise stated

*In vitro* group	Broad annotation	Top mimic drugs	Target	Score
6	Cytokine	Ingenol 3, 20-dibenzoate	PKC activator	0.0534
33	HDAC inhibition	Vorinostat	HDACs	0.0933

Disease Group		Top reverse drugs	Nominal target	Score

8	RA	Curcubitacin I	JAK/STAT3	0.0389
		15d-PGJ2	PPARG activator	0.0354
		Bortezomib	Proteasome	0.0349
11	Osteoarthritis	Narciclasine	Apoptosis	0.0659
		Manumycin A	Ras	0.0579
		Salermide	SIRT1/2	0.0568
35	Animal models Osteoarthritis	Selumetinib	MEK1/2	0.0574
		TG101348	PI3K	0.0549
		BMS-536924	IGF1R	0.0544

### 3.4 SkeletalVis web portal

#### 3.4.1 Exploration module

To enable future exploration and comparison of these data we constructed an interactive data-portal, SkeletalVis. SkeletalVis is composed of exploration and comparison modules as well as a detailed help section (Supplementary Fig. S4a). The exploration section of the data portal allows visualization of the detailed analysis (Supplementary Fig. S4b). To illustrate the utility of the exploration module we selected a well-characterized experiment investigating the expression profile of a Col10a1 knock-in mutation mouse, which is a model of the Metaphyseal chondrodysplasia type Schmid (MCDS) form of dwarfism (GSE30628) (Cameron *et al.*, 2011). An experimental table shows the available experiments with the ability to sort, search and filter the table to find an experiment of interest (Supplementary Fig. S4c). From a chosen experiment the user can view and then select a comparison associated with that experiment to load the data in the other tabs (Supplementary Fig. S4d).

**Fig. 3.**
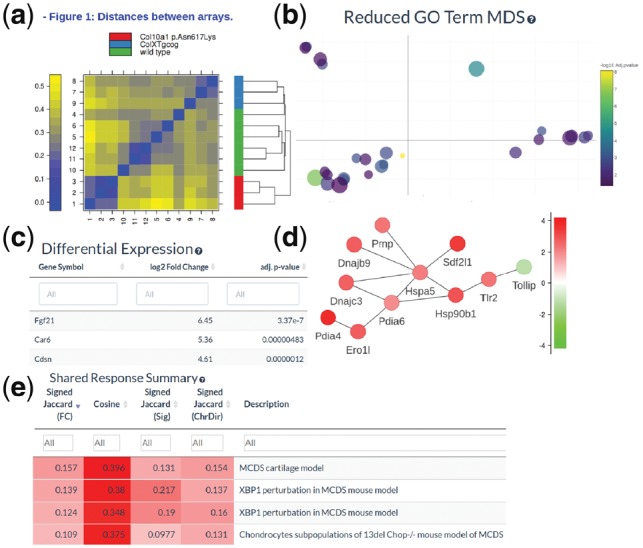
Analysis of MCDS mouse model with SkeletalVis. SkeletalVis provides quality control (**a**), differential expression analysis (**b**) with detailed downstream analysis including GO term enrichment (**c**) and network analysis (**d**). The shared gene expression responses can be readily examined (**e**)

Once an experiment and comparison are loaded, quality control summaries including heatmaps allow the user to quickly assess the quality of the data (Fig. 3a). The data portal allows searching of the differential expression table with fold-changes and adjusted *P*-values (Fig. 3b). This table can be searched to find particular genes and can be filtered with a user defined thresholds to identify differentially regulated genes. All tables in the data portal can be copied or exported as text files for use with external tools. SkeletalVis provides detailed downstream analysis with enriched pathways, drugs, transcription factors, which can be viewed in interactive tables to identify the key dysregulated biological processes. As GO enrichment is often difficult to interpret, we use interactive multi-dimensional scaling plots based on the semantic similarity of the ontology terms to group-related terms together allowing a quick overview of the perturbed processes (Fig. 3c). Active sub-networks can be viewed as interactive networks coloured by fold change which often give more sensitive analysis compared with standard pathway enrichment analysis (Fig. 3d). For the MCDS experiment pathways, transcription factors and active sub-networks relating to endoplasmic reticulum stress and the Atf4 transcription factor are consistent with the findings reported in the corresponding publication.

The shared response tool shows pre-calculated expression similarities to every other comparison in the data portal using the four above described measures. This module is of use for both quality assurance in ensuring the expression response is similar to a related experiment, and for discovery of novel associations between disease and genetic/exogenous perturbations. The genes overlapping with each expression measure are shown and histograms show the distribution of the similarity scores. In the MCDS comparison data, among the top hits is an independent profile from mice with the same causative mutation knock-in and a profile from an alternative transgenic mouse model in the same causative Col10a1 gene, allowing consensus signatures to be developed (Fig. 3e).

#### 3.4.2 Comparison module

The comparisons module allows for comparison of newly generated data to the experiments analysed in SkeletalVis. Gene signatures (up- and down-regulated genes) identified from an experiment outside the data portal can be compared with the differential expression and characteristic direction signatures within the portal. Overlap of the genes between the signatures is shown to allow focus on shared genes, allowing identification of novel associations between datasets. To illustrate the utility of this module the gene expression signature reported in the recent paper examining bone lesions in osteoarthritis was queried against the signatures in the data portal (Supplementary Fig. S5) (Kuttapitiya *et al.*, 2017). Among the most similar experiments is a study on subchondral bone in an osteoarthritis surgical mouse model suggesting that cross-species similarities in the signatures can be observed. The module highlights the overlapping genes including THBS4 known to be involved in pain sensitization which is highlighted in the corresponding publication. The comparison module can also be used identify experiments where a particular gene is dysregulated, to identify exogenous perturbations that modulate the expression of that gene or finding other diseases where that gene is dysregulated. For example, searching for THBS4 reveals human osteoarthritis datasets where THBS4 is also up-regulated illustrating how SkeletalVis allows rapid cross-species comparison of newly available gene expression responses against this existing repository of knowledge.

## 4 Discussion

With the expanding expression data available for the study of skeletal disease, SkeletalVis collates 287 cross-species skeletal transcriptomic experiments and is an intuitive data-portal to allow exploration and meta-analysis. This consolidation of complex data to an accessible format is crucial to gaining meaningful information from the large numbers of datasets. Through our analysis of gene perturbation response associations we have highlighted several examples of links between cross-species *in vitro* and *in vivo* experiments. For instance, our findings suggest that modulation of the polycomb repressive Complex 2 role could be targeted to modulate the chondrocyte de-differentiation gene expression signature that occurs with culturing chondrocytes for future cell therapy to treat cartilage degeneration (Ma *et al.*, 2013). The comparisons of expression profiles from the SCFE model, Hyp and hormone signalling perturbations have not been previously reported but AHSG is a known transcriptional target of ERα and these findings are consistent with suggestions it is a hormonal balance driven skeletal disorder (Qiu *et al.*, 2014; Witbreuk *et al.*, 2013).

We identified groups of similar signatures and identified enriched drug responses in consensus signatures to demonstrate the ability of this analysis to find regulators of core differentially expressed genes in groups of transcriptomic responses. Although the drug response signatures are derived from treated cancer cell lines rather than skeletal cell types, several of the highlighted top drug predictions are support by previous studies. In the RA group, the predicted reverse drug targets JAK/STAT3 and the proteosome are known inflammatory mediators (Elliott *et al.*, 2003; Schwartz *et al.*, 2017). PPARγ has previously been suggested as a potential therapeutic target in RA (Ormseth *et al.*, 2013). In the model OA group, pharmacological MEK and PI3K inhibition protected against cartilage damage in rabbit and mouse OA models respectively (Lin *et al.*, 2018; Pelletier *et al.*, 2003). In the OA cartilage group, the enriched drug narciclasine reduced joint destruction in a rat model of arthritis (Lubahn *et al.*, 2012). Interestingly, Salermide a Sirt1/2 inhibitor is an enriched reverse drug in the OA cartilage group consensus signature and in several individual studies examining human intact OA versus non-OA cartilage within that group. Evidence from *in vivo* mouse studies suggests that that Sirt1/2 activity is protective in OA (Matsuzaki *et al.*, 2014). This expression overlap may therefore represent activation of the protective Sirt1/2 pathway in the intact OA cartilage. These data therefore allow development of many hypotheses to be followed up with new data and functional studies.

The aim of SkeletalVis is not to replace existing tools such as ExpressionAtlas, but to provide a more specialized repository for skeletal disease researchers with extended downstream data analysis. For example, searching for the common skeletal disease osteoarthritis in ExpressionAtlas returns only 3 experiments compared with 30 in SkeletalVis. Several existing cancer expression specific databases such as GlioVis focus on integration of survival and somatic mutation data with expression data which is not generally applicable for skeletal diseases (Bowman *et al.*, 2017). Instead, SkeletalVis offers added value in coverage of skeletal datasets and considerably more in-depth down-stream analysis with network analysis and integration with databases such as LINCS L1000 and also includes the ability to compare expression profiles. This data portal is of wide use to skeletal biology/disease researchers as it can be used to rapidly screen for evidence of target gene dysregulation in skeletal development and disease, and also to identify perturbations that can be modulated to altered expression activity of these targets in a skeletal cellular context. The portal will be useful for prioritization of identified differentially expressed genes for experimental validation and for initial functional characterization of novel disease-associated genes identified through genome-wide association studies, allowing understanding of potential functions of the genes in context of skeletal tissues. Not all included studies have sufficient replicates to calculate the statistical significance of the altered expression. Although these studies can be readily filtered from the tables, these studies often describe unique perturbations in the database and may be useful for researchers interested in finding shared perturbations for further experimental validation. Curation efforts to make a context-specific database are likely to produce more immediately relevant results for users than generic databases. Although gene expression responses can be shared with non-skeletal tissues, the specialized nature of the skeletal tissues makes investigating perturbations within the same biological system more useful. The approach herein is likely to be of interest to many investigators building context-specific omics databases. The developed pipeline and app can be deployed in other areas of biological interest and this collection of data with known perturbations will be useful for development and validation of methods for analysing skeletal transcriptomics data.

As new expression data becomes available in public repositories this data can readily be analysed and integrated into the web platform. Similarly, as new methods of analysis are developed these can be performed on these collection of datasets. Future updates could include integration of further omics data such as non-coding transcription, proteomics and epigenetic data so to investigate the interplay of multiple regulatory layers.

## Supplementary Material

bty947_Supplementary_Data
