# Supplementary material for: SkeletalVis: an exploration and meta-analysis data portal of cross-species skeletal transcriptomics data
Source: Bioinformatics. 2018 Nov 27;35(13):2283–90. doi: 10.1093/bioinformatics/bty947 (PMC6596879; doi:10.1093/bioinformatics/bty947)
Supplement: bty947_Supplementary_Data [file bty947_supplementary_data.zip › SupplementryFigures.docx]

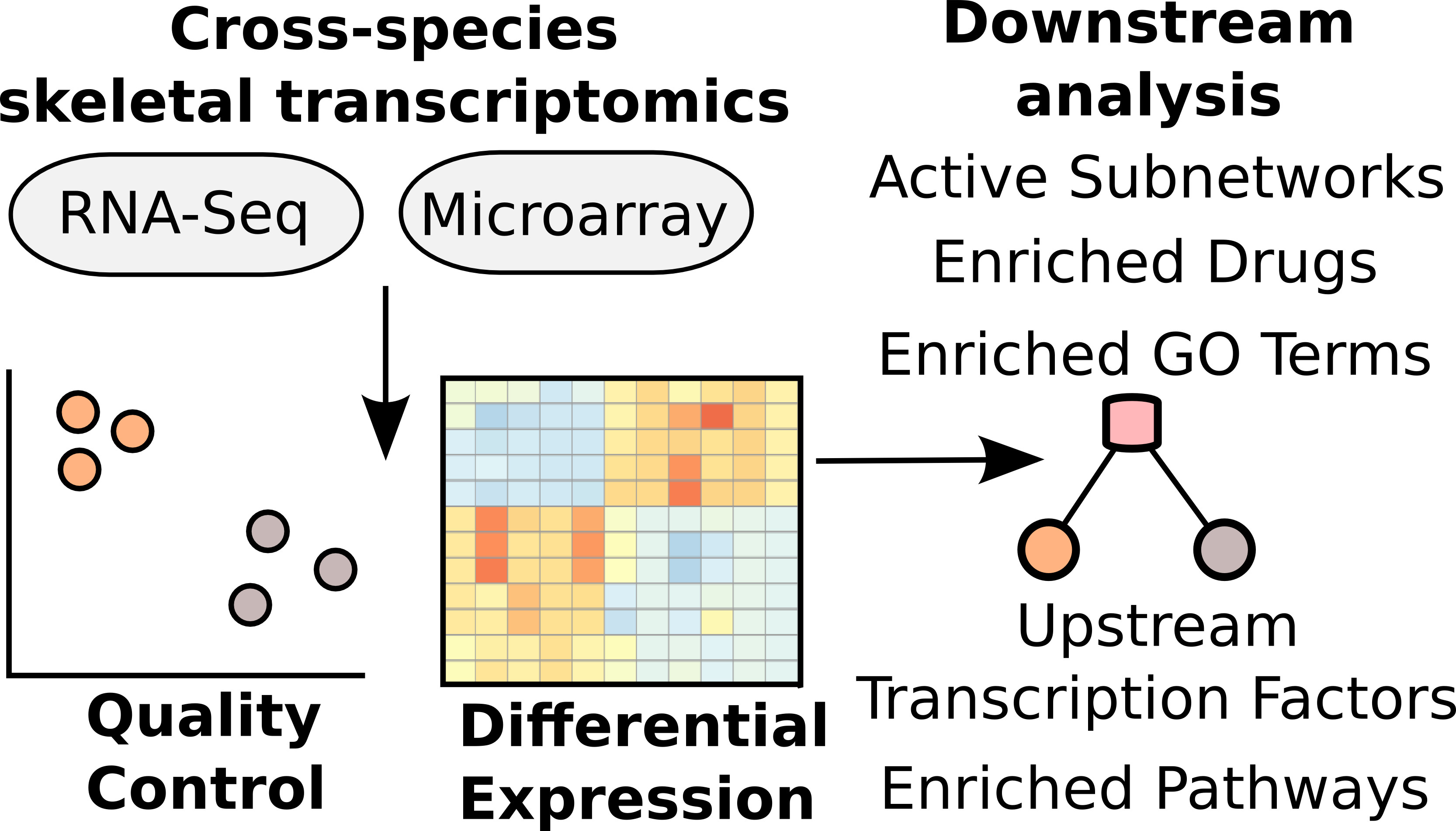


1. **Supplementar****y Fig 1: Schematic diagram of the SkeletalVis transcriptomics pipeline**
2.
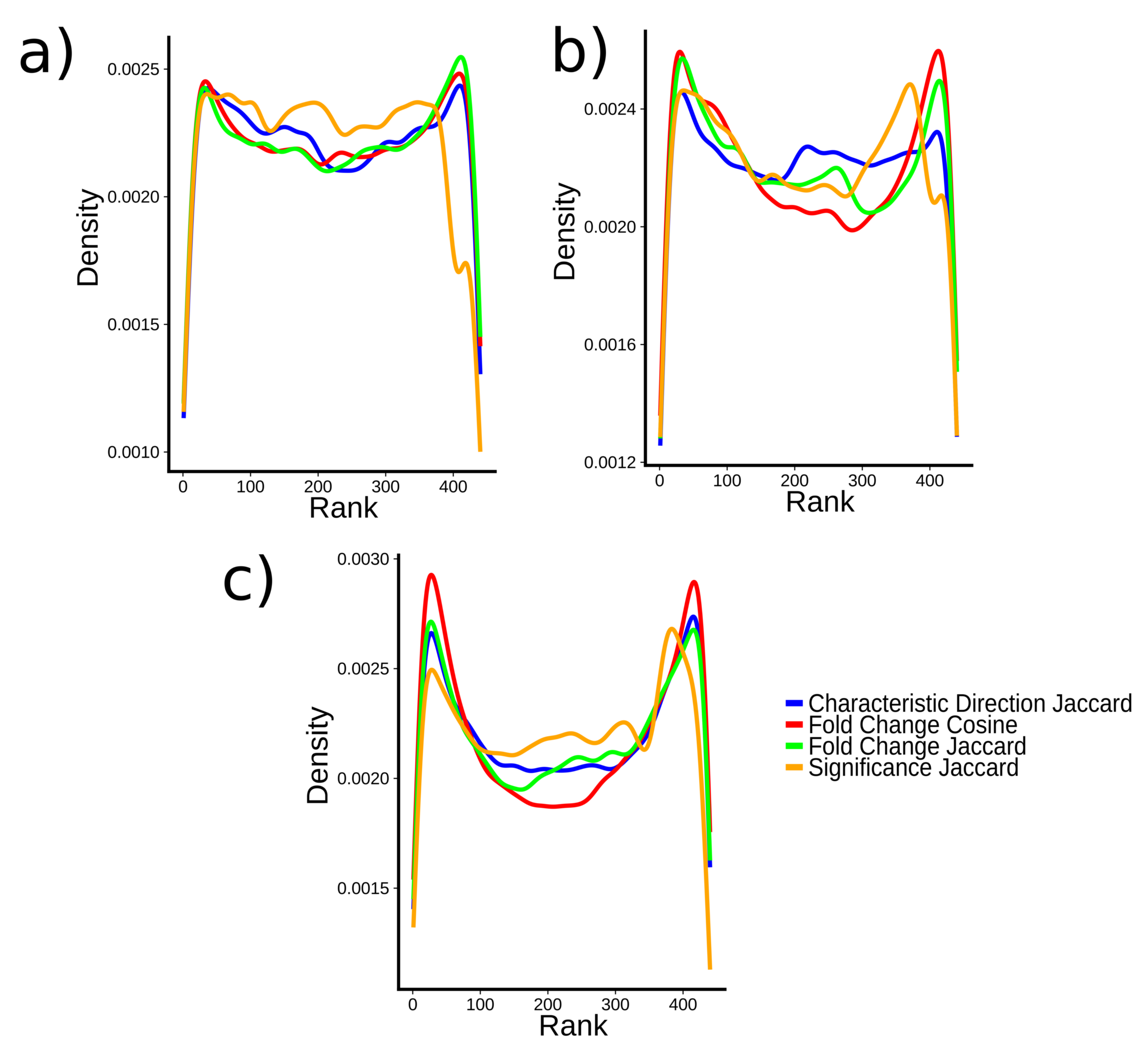

3. **Supplementary Fig 2: Assessment of bias in the gene expression signature similarity**
4. The rank of signature similarity was determined using four gene expression similarity measures for experimental perturbations within the same experimental platform (a), tissue of origin (b), and species (c).
5. **
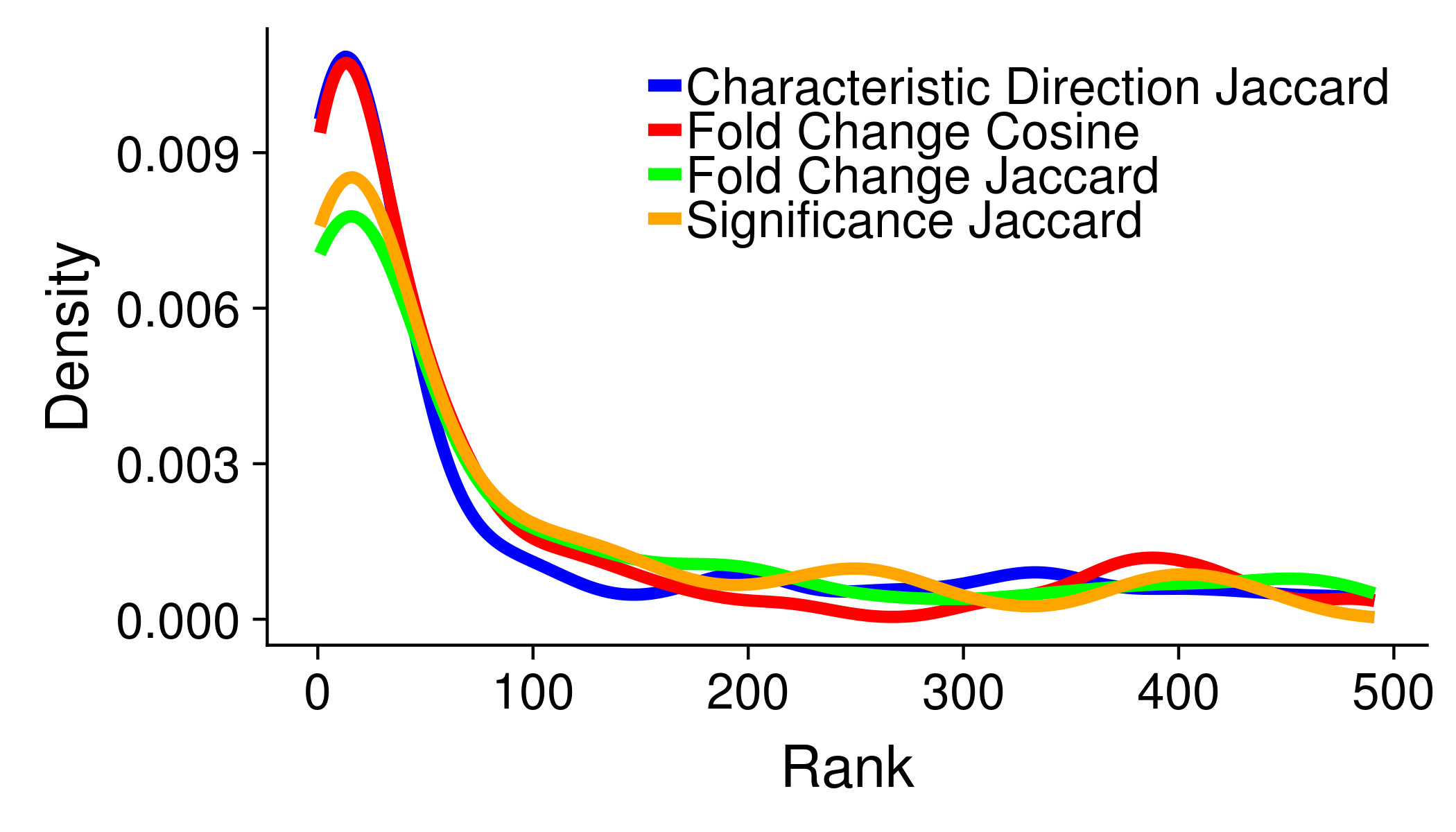
**
6. **Supplementary Fig. 3 Validation of expression similarity measures**
7. The rank of gene expression signature similarity was determined using four similarity measures for sets of related experimental perturbations.
8. **
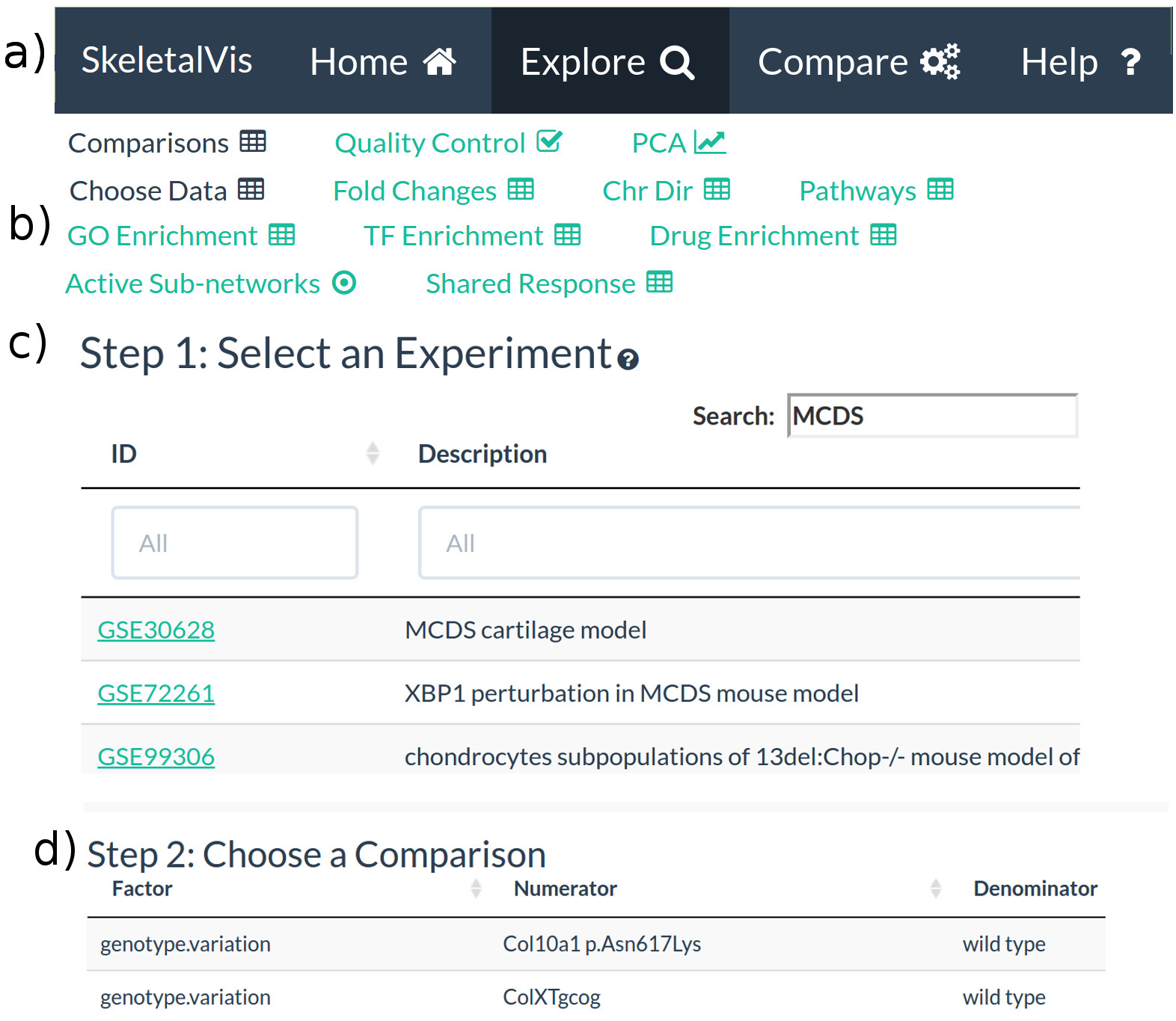
**
9. **Supplementary Fig 4: Exploration of experiments in SkeletalVis**
10. The SkeletalVis menu bar allows selection of the exploration, comparison and help modules (a).The exploration module gives comprehensive analysis of the transcriptomic datasets (b) Users can view the available experiments in an interactive table (c) and select a comparison of choice (d).
11.
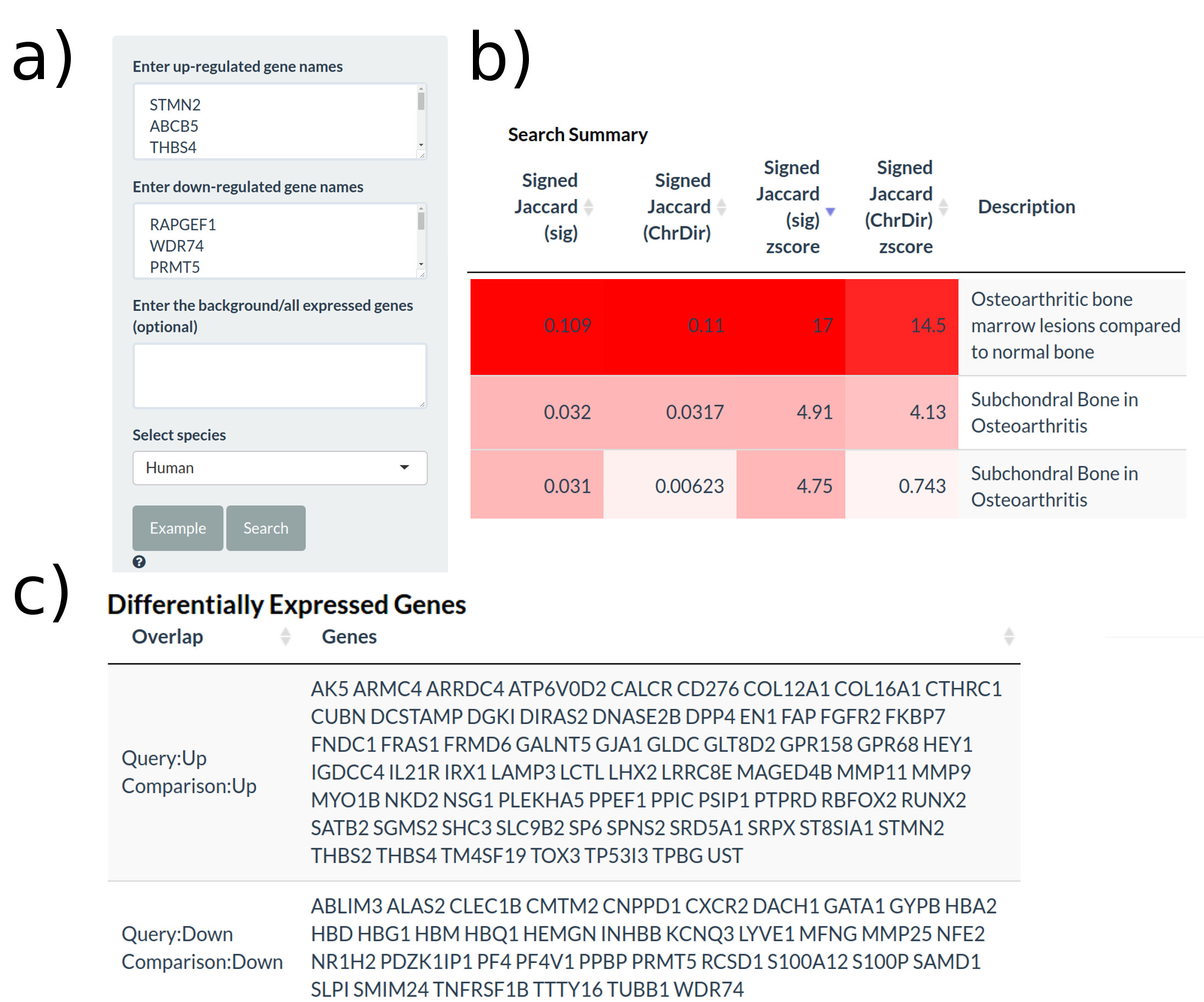

12. **Supplementary Fig 5: Comparison of user-provided signatures in SkeletalVis**
13. The gene symbols of up- and down- regulated genes can be entered in the text boxes along with the species (a). The entered expression signature is compared to all other signatures in the data portal and displayed in a summary table (b). Selection of a row in the search summary table gives the genes that are overlapping between the input and selected signatures (c).
